# Systemic treatment of advanced/metastatic renal cell carcinoma in the context of SARS-CoV-2 pandemic: recommendations from the interdisciplinary working group for renal tumors (IAG-N)

**DOI:** 10.1007/s00432-020-03341-4

**Published:** 2020-08-20

**Authors:** Philipp Ivanyi, Carsten Grüllich, Nils Kroeger, Thomas Gauler, Manfred Johannsen, Jens Bedke, Viktor Grünwald, On behalf of the Interdisciplinary working group on renal tumors (IAG-N) of the German Cancer Socity (DKG)

**Affiliations:** 1 Department of Hematology, Hemostasis, Oncology and Stem Cell Transplantation, Hannover Medical School, Hannover, Germany; 2 Immunonclogical COoperative Group (ICOG) of the Comprehensive Cancer Center Lower Saxoney, Hannover (CCC-N), Hannover, Germany; 3 Genitourinary Oncology, Department of Urology, University Hospital Dresden, Dresden, Germany; 4 Department of Urology, University Medicine Greifswald, Greifswald, Germany; 5 Urologie-Altstadtquartier Magdeburg, Magdeburg, Germany; 6 Department of Radiation Oncology, West German Cancer Center, University Hospital Essen of the University Duisburg-Essen, Essen, Germany; 7 Urology and Urooncology Practice Berlin, Vice Chairman d-uo (Deutsche Uro-Onkologen), Berlin, Germany; 8 Department of Urology, Eberhard Karls University Tuebingen, Hoppe-Seyler-Str. 3, 72076 Tübingen, Germany; 9 Interdisciplinary Genitourinary Oncology, West-German Cancer Center Essen Clinic for Medical Oncology and Clinic for Urology OZ1, University Hospital Essen (AöR), Room 2.073 Hufelandstraße 55, 45147 Essen, Germany

## Abstract

This letter summarizes recommendations from the interdisciplinary working group of renal tumors (IAGN) of the German Cancer Society for the systemic treatment of advanced/metastatic renal cell carcinoma in the context of the current SARS-CoV-2 pandemic

## Background

During the SARS-CoV-2 pandemic, oncologists faced several new challenges (Jones et al. 2020; Hanna et al. 2020; Lai et al. 2020). Data on SARS-CoV-2 in cancer patients suggested outstanding rates of severe clinical courses (39–75%) and mortality (up to 29%) upon SARS-CoV-2 infection, although methodological limitations of these observations are of concern (Desai et al. 2020; Liang et al. 2020; Yang et al. 2020; Zhang et al. 2020). However, oncological therapies might raise SARS-CoV-2-related morbidity and mortality (Desai et al. 2020; Liang et al. 2020; Yang et al. 2020; Zhang et al. 2020). Checkpoint inhibitors (CPI) could negatively interfere with the pathogenesis of SARS-CoV-2, and an overlap of symptoms of SARS-CoV-2 and cancer treatment related adverse events can be assumed which could result in a diagnostic challenge (Zhang et al. 2020; Bersanelli 2020; Rotz et al. 2017). In particular, interstitial pneumonia from SARS-CoV-2 cannot be differentiated from CPI-associated pneumonitis with certainty using CT images, and may present with overlapping features (Zhang et al. 2020; Bersanelli 2020). Pneumonitis in CPI-treated patients are rare with an incidence von 2–10% but accounts with treatment-related death rate of 35% (Wang et al. 2018; Naidoo et al. 2017). Effects of immune suppression due to supportive measure has also to be considered, although only preliminary data exist on effects of steroids in cancer patients during the SARS-CoV-2 pandemic (Yang et al. 2020). As a result, use of CPI in advanced/metastatic renal cell carcinoma patients (mRCC) may be associated with a specific risk, and requires a critical risk–benefit assessment. Therefore, choosing the appropriate therapeutic regimen in mRCC is more challenging than ever.

Several recommendations focus on reducing the risk of SARS-CoV-2 exposures for patients, as well as for medical staff, as part of oncological routine and addresses mainly questions of resource allocation. Discussion about treatment-specific considerations remains scare. Herein, we report a consensus of the interdisciplinary working group on renal tumors (IAG-N) of the German cancer society towards treatment of mRCC.

During the process of treatment evaluation, the assessment of treatment indication must be made on an individual basis. However, once selecting treatment, patient, and tumor-specific parameters, patient´s comorbidity and the availability of local caregiving facilities should be taken into account. In our opinion, two major scenarios according to systemic treatment have to be considered: initiation or change medication of a systemic treatment upon progressing diseases and measures taken during ongoing systemic treatment.

## Considering urgency once initiating treatment upon advanced or metastatic RCC

For systemic treatment, the appropriate treatment should be selected based on patient-specific factors that take the overall picture into account when searching for treatment (Escudier et al. 2019). The indication for the systemic treatment of mRCC should be made strictly, taking active surveillance and deferred medical treatment into consideration (Escudier et al. 2019). This should minimize patient exposure to the at-risk medical care environment, as well potentially towards particular treatment-associated risk in terms of SARS-CoV-2 infection. Patients who do not require urgent systemic treatment should, therefore, primarily be offered active surveillance and deferred medical treatment. No validated tool that identifies indolent disease is established. However, various parameters are used by clinicians to identify the appropriate patient population for active surveillance. Table 1 represents a number of such variables, which are used by the authors.

**Table 1:** Clinical parameters to estimate the urgency of treatment need in patients with metastasized renal cell carcinoma

Parameters	Favorable	Unfavorable
Patient-related	Oligometastasis glandular involvement^a^ 1 Organ system involved asymptomatic ECOG: 0–1	High tumor load polytopic bone metastases and/or multiple organ systems ECOG 2 Risk factors for secondary complications^b^
Tumor-related	Clear cell RCC	Sarcomatoid subtype, medullary subtype, non-clear cell subtypes
Prognosis according to the IMDC	Favorable risk	Intermediate and poor risk

^a^Pancreas, thyroid gland, saliva, adrenal glands

^a,b^Impending complications in the event of further progression (e.g. bronchial compression, pathological fracture, cross section, vascular erosion/occlusion)

Also, considering the CARMENA-discussion on palliative nephrectomy, in asymptomatic synchronous metastasized patients, cytoreductive nephrectomy should be considered critically, respecting the fact of reported nosocomial SARS-CoV-2 infection in cancer patients (28.6%), as well as a surprising high rate of SARS-CoV-2 diseases among cancer patients receiving surgery (Liang et al. 2020; Zhang et al. 2020).

## How should I treat a patient with favorable risk?

Suitable systemic treatment is selected based on its marketing authorization, principle recommendations, as well as on published data. The strengths and weaknesses of a treatment regime should be weighed against its suitability for the best possible application. In order to minimize the treatment risk and if the clinical course is indolent, the necessity for systemic therapy is low and active surveillance is our preferred option. Re-staging is recommended in 2–3 months’ time. Based on these considerations, the feasibility of an active monitoring strategy or initiation of a therapeutic measure should be evaluated.

In light of CheckMate214, JAVELINRenal 101 and Keynote-426 studies, no significant overall survival (OS) benefit was gained by any of the immune combinations for patients with a favorable risk profile (HR for OS: ipilimumab/nivolumab vs. sunitinib: 1.19 (95% CI 0.77–1.85), axitinib/pembrolizumab vs. sunitinib: 0.94 (95% CI 0.43–2.07), axitinib/avelumab vs. sunitinib: 0.812 (95% CI 0.336–1.960) (Motzer et al. 2020; Keytruda-EMEA 2020; Choueiri et al. 2020). A major limitation of these data are the short follow-up duration, which limits the data interpretation. However, at the current state, there is no signal that a specific combination is superior in OS expectations when compared to single agent sunitinib. This is supported by molecular findings, wherein IMDC favorable risk is associated with pro-angiogenic dependency (McDermott et al. 2018). However, a proportion of patients exert an inflamed tumor type, which may identify a patient population with potential clinical benefit from CPI treatment. Today, there is no clinical test available to identify such patients. The best treatment strategy in these patients’ remains has not been defined and data is scarce. The identification of indolent disease and the absence of a survival signal for combinations in this patient population should caution treatment intensification and puts active monitoring and single agent TKI as preferred treatment options in the treatment algorithm of this patient cohort.

## How should I treat a patient at intermediate or poor risk?

In cases who exert favorable clinical parameters depicted Table 1, patients with intermediate risk may receive active monitoring and deferred medical treatment as an alternative strategy to upfront medical treatment. However, most patients require systemic treatment and clinical benefits outweighs risk of immune-related adverse events/SARS CoV-2.

Patients with an intermediate or unfavorable risk profile had longer OS when taking immune combinations than patients taking TKI alone, which is why they should be used preferentially [HR for OS: ipilimumab/nivolumab vs. sunitinib: 0.66 (95% CI 0.55–0.8); axitinib/pembrolizumab intermediate prognosis: 0.53 (95% CI 0.35–0.82), poor prognosis: 0.43 (95% CI 0.23–0.81); axitinib/avelumab: intermediate prognosis: 0.86 (95% CI 0.615–1.202), poor prognosis: 0.57 (95% CI 0.363–0.895); Cabozantinib vs. sunitinib: 0.80 (0.53–1.21)] (Motzer et al. 2020; Keytruda-EMEA^2020^; Choueiri et al.  2018, 2020). To minimize the risk of immune-mediated adverse events, as well as high dose steroids application due to immune related adverse events, a careful patients based valuing is demanded to choose the best treatment regimen (Table 2). Overall, if clinical parameters in this cohort are favorable, the TKI cabozantinib reflects a therapeutic option. Otherwise, an immune combination represents the preferred form of treatment, in particular in poor prognosis patients, wherein the clear harm of the mRCC and the oncological efficacy overweight’s the risk of pandemic associated concerns.

**Table 2:** Summary of the treatment recommendations for first-line treatment of metastatic RCC during the SARS-CoV-2 pandemic

IMDC risk groups	1. Selection	Option
Favorable	Active surveillance	TKI
Intermediate	Axitinib + avelumab Axitinib + pembrolizumab	Active surveillance (restaging in 3 months) cabozantinib Ipilimumab + nivolumab
Unfavorable	Axitinib + avelumab Axitinib + pembrolizumab Ipilimumab + nivolumab	Cabozantinib

The list is shown in alphabetical order. When selecting treatment, the individual patient and tumor characteristics listed in Table 2 should be considered

## What to conclude in SARS-CoV-2 pandemic for mRCC

Although precise guidelines according to mRCC treatment reflects best efficacy and QoL data, within the SARS-CoV-2 pandemic a patient-centered treatment choice, which is adapted to the local pandemic situation is warranted (s. Table 2). Reflecting SARS-CoV-2-related comorbidity, patient´s and tumor characteristic´s, adverse events and hospitalization rates seem to be useful parameters to adjust risk/benefit ratio pandemic-adapted mRCC treatment choice. However, expanding real-world register data will answer the question which concerns where the right one, over- or under-treatment.
